# Analgesia and Sedation of Pediatric Patients with Major Trauma in Pre-Hospital and Emergency Department Settings—A Narrative Review

**DOI:** 10.3390/jcm12165260

**Published:** 2023-08-12

**Authors:** Neta Cohen, Daniel M. Cohen, Egidio Barbi, Itai Shavit

**Affiliations:** 1 Pediatric Emergency Medicine Department, Dana Dwek Children’s Hospital, Tel Aviv Sourasky Medical Center, 6 Weizman St, Tel Aviv 6423906, Israel; 2 Faculty of Medicine, Tel Aviv University, Tel Aviv 6997801, Israel; 3 Nationwide Children’s Hospital, The Ohio State University, Columbus, OH 43210, USA; daniel.cohen@nationwidechildrens.org; 4 Department of Pediatrics, Institute for Maternal and Child Health—IRCCS Burlo Garofolo, 34137 Trieste, Italy; egidio.barbi@burlo.trieste.it; 5 Clinical Department of Medical Surgical and Health Science, University of Trieste, 34127 Trieste, Italy; 6 Division of Pediatrics, Hadassah Medical Center, Jerusalem 9112001, Israel; shavit1@hadassah.org.il; 7 Faculty of Medicine, The Hebrew University of Jerusalem, Jerusalem 9112102, Israel

**Keywords:** pediatric patients, major trauma, analgesia, sedation, emergency department, pre-hospital care

## Abstract

Children who sustain major injuries are at risk of receiving insufficient pain relief and sedation, which can have physical and psychological repercussions. Heightened emotional distress can increase the likelihood of developing symptoms of post-traumatic stress. Providing sufficient analgesia and sedation for children with major trauma presents specific challenges, given the potential for drug-related adverse events, particularly in non-intubated patients. The current literature suggests that a relatively low percentage of pediatric patients receive adequate analgesia in pre-hospital and emergency department settings following major trauma. There are only sparse data on the safety of the provision of analgesia and sedation in children with major trauma in the pre-hospital and ED settings. The few studies that examined sedation protocols in this context highlight the importance of physician training and competency in managing pediatric airways. There is a pressing need for prospective studies that focus upon pediatric major trauma in the pre-hospital and emergency department setting to evaluate the benefits and risks of administering analgesia and sedation to these patients. The aim of this narrative review was to offer an updated overview of analgesia and sedation management in children with major trauma in pre-hospital and ED settings.

## 1. Introduction

The importance of appropriate acute pain management in children, especially following traumatic injuries and during painful procedures, is well-recognized [1,2,3]. Early and adequate provision of analgesia and sedation is a fundamental component of quality patient care in emergency medicine, given that failure to do so can result in short- and long-term physical and psychological sequelae, especially in children [4,5,6,7]. Inadequate pain control following an injury can manifest in acute physiologic, biochemical, and behavioral sequelae [7], and may result in stress reactions that can lead to the development of persistent post-traumatic stress symptoms (PTSS) [8]. In their prospective longitudinal study, Hildenbrand et al. [9] reported that severe pain was a significant predictor of both concurrent and later PTSS in children who sustained trauma. Those authors observed that acute pain levels were able to predict the presence of PTSS six months after the injury, even after accounting for other potential risk factors. Similarly, evidence from studies on adults who sustained traffic-related and burn injuries also suggested an association between pain and the development of PTSS [8,9].

The evaluation of pain levels in the trauma patient poses unique challenges as it may involve both somatic and visceral pain from a variety of origins. Greenwald et al. [7] reviewed the nature of pain with respect to its pathophysiology, clinical ramifications, and patterns of analgesia practices in injured children. Those authors described various reasons for poor pain management, stemming from either inadequate pain assessment skills, lack of understanding about pain and analgesics, and excessive concern regarding hemodynamic fluctuations and respiratory depression [7]. The fear of masking signs of serious injury has also been described in the literature [7,10]. For example, one study that included 215 physicians and nurses from nine trauma units reported that analgesics were frequently (78%) withheld to “assist diagnosis” [10]. Several other studies, however, found no correlation between the appropriate provision of opioids and missed injuries or delayed imaging/diagnosis [11,12].

Notably, most children with major trauma are not treated with tracheal intubation during pre-hospital and emergency department (ED) care [13]. Providing sufficient analgesia and sedation in these patients poses specific challenges due to concerns for potential drug-related adverse events in the non-intubated patient, such as respiratory depression and hypotension [13].

There are sparse data on the safety of the provision of analgesia and sedation in children with major trauma in the pre-hospital and ED settings. Of note, the advanced trauma life support (ATLS) student manual [14], which serves as the accepted reference for managing patients with major trauma, does not include a dedicated chapter on pain and sedation management. The few paragraphs throughout the manual that do address these issues emphasize the need for cautious and minimal use of opioids or anxiolytics to mitigate the risk of adverse events.

The aim of this narrative review was to offer an updated overview of analgesia and sedation management in children with major trauma in pre-hospital and ED settings.

## 2. Methods

### 2.1. Search Criteria

#### 2.1.1. Type of Studies

We included original research, review articles, meta-analysis, survey studies and books of any date in which sedation/analgesia was performed on children following major trauma in the pre-hospital or in the ED setting, as defined below. Relevant studies were chosen based on the following criteria: (1) the study population involved children under the age of 18 years; (2) the patients presented to the ED following major/multisystem trauma, as defined in Section 2.2; (3) the study deals with pain management and/or in procedural sedation and analgesia (PSA). Additional inclusion criteria were: (1) the article was published in English; (2) Peer-review format; (3) could be obtained via journal access from the host institutions (Tel Aviv sourasky medical center and Tel Aviv University). Final decisions to include studies were made by agreement between the authors.

#### 2.1.2. Search Strategy

We conducted a comprehensive search of electronic databases, including Ovid Embase, Scopus, PubMed and the Cochrane Database of Systematic Reviews. The internet search engine Google Scholar was searched using the search terms: Children, Pediatric patients, Pre-hospital, Emergency medical service (EMS), Helicopter emergency medical services (HEMS), Aeromedical Retrieval Service (AMRS(, Multi-system trauma, Major trauma, Trauma room, Trauma-bay, Injury, Pain management, Opioids, Fentanyl, Ketamine, Midazolam, Methoxyflurane, Propofol, Procedural sedation and analgesia (PSA), and Emergency department (ED), to identify articles published in electronic journals, books and scientific websites. We also completed a hand search of references of the included studies.

### 2.2. Definitions of Major Trauma

Major trauma refers to severe and life-threatening injuries, often resulting from accidents or acts of violence. It involves physical injury that can potentially cause long-term disability or death. While there is no universally agreed-upon definition, major trauma typically includes injuries, such as severe head trauma, multiple fractures, spinal cord injuries, severe burns, organ damage, and injuries requiring immediate surgical intervention [14,15]. The definitions of major trauma in the literature are inconsistent between studies and based upon a number of measurements, including the Injury Severity Score (ISS) or the need for trauma team activation [13,16,17,18,19]. In addition, studies that defined major trauma by the ISS used different cut-off values [13,16,19].

#### 2.2.1. Pre-Hospital Study Requirements

For this narrative review, we included studies on pre-hospital pain and sedation management in the pediatric population which fulfilled at least one of the following criteria:

1. Patients transported via the aeromedical services, which are typically deployed to transfer severely injured children [16,20,21,22,23,24,25].
2. Patients selected by injury severity defined by either ISS, admission to an intensive care unit, or in-hospital death [14], even if they were transported by ground ambulance.
3. Patients who were treated by military medical providers in a combat setting [26].

#### 2.2.2. ED Study Requirements

We included ED studies on pediatric patients whose injury was defined as being severe either by ISS [13,19], first-tier trauma team activation [17], or those who met the American College of Surgeons Trauma Center Guidelines [18].

## 3. Results and Discussion

### 3.1. Adequacy of Pain Management in Major Trauma Patients

Various patient and system-level factors were associated with the adequacy of pain management. Numerous studies found young age to be associated with inadequate analgesia [17,18,20,25], regardless of the setting (pre-hospital or ED). Neighbor et al. [17] observed that young children (≤10 years) received significantly fewer narcotic pain medications in the ED compared to adults and older children. Rugg et al. [20] reported that intravenous access was less often established in children <6 years of age, and that they were less likely to receive esketamine compared to older children (>10 years) in transport via the Helicopter Emergency Medical Services (HEMS).

Several studies examined the association between the severity of trauma and the administration of opioid analgesia [16,17,18,19,25,26]. Interestingly, some of those studies showed that patients with more severe trauma, as indicated by a higher ISS, or by a greater number of interventions, received significantly greater amounts of narcotic analgesia [17,27,28]. Notably, however, other studies have reported contradictory findings, and suggested that patients with more severe trauma are less likely to receive opioid analgesia [16,17,18].

Anantha et al. [19] explored the use of analgesia of severely injured children in the ED during the resuscitative phase and found that activation of the trauma team and direct transport to a trauma center were associated with higher rates of analgesia compared to initial presentation to a peripheral hospital [19]. Importantly, the presence of parents in the resuscitation room emerged as a significant predictor for analgesia usage during early resuscitation [19].

With respect to injury mechanisms, studies observed that motor vehicle collisions [19] and explosive injuries [26] were associated with higher odds of analgesia administration rates.

### 3.2. Pre-Hospital Analgesia for Children with Major Trauma (Table 1)

As noted above, pre-hospital studies were included in this review if they involved pediatric patients who were transported by aeromedical services [20,21,25] or by ground ambulances, if the study defined major trauma by other measurements (ISS, admission to an intensive care unit, or in-hospital death) [16], or children treated by military medical personnel in a combat setting [26]. Overall, there was a wide range of analgesia administration rates (15–52%) for such major trauma patients. Barker et al. [21] explored 349 pediatric patients who had been treated by an Australian HEMS and found a 15% administration rate of analgesia. Rugg et al. [18] investigated the provision of analgesia to injured children who were transported via an Austrian HEMS and reported that analgesic medications were administered in 31.4% (3874 of 12,324 patients) of all cases. Johnson et al.’s [25] survey of an air medical transport agency in the United States showed that only 14.6% of 5057 severely injured patients received analgesia. One possible explanation for that low rate was attributed to the fact that only 39% of the patients had any pain score documentation. The authors mentioned that more than 60% of the children who did have documentation of severe pain (≥5/10) received analgesics. Curtis et al. [16] conducted a study on pre-hospital pain treatment-related elements in pediatric patients with major trauma in Australia who were transported by either ground ambulances or aeromedical services. Seventy-seven percent of the patients (279/359) received analgesia. Among them, opioids were provided to 157 (52%) children.

**Table 1:** Medication dosages administered for pre-hospital analgesia and sedation among children who sustained major physical trauma.

Medication	Mean Dosage	Country	Study	Comments
Fentanyl	0–15 years: 0.33–5.0 microgram/kg	USA	DeVellis et al. [22]	Fentanyl was given for analgesia during air transport.
	0–5 years: 0.05 mg (0.03–0.07)	Austria	Rugg et al. [20]	Opioids were given for analgesia.
	6–10 years: 0.05 mg (0.05–0.10)			
	11–14 years: 0.10 mg (0.05–0.20)			
	0–18 years: 1–3 microgram/kg per dose with maximum hourly dose of 7 microgram/kg (in intubated patients) or 5 microgram/kg (in non-intubated patients)	USA	Thomas et al. [23]	Fentanyl was given for analgesia. Per protocol, “Hypotension is much less of a problem with fentanyl than with other opioids, but any analgesic should be administered with caution in patients who are at risk for hemodynamic deterioration.”
Piritramide	0–5 years: 2.38 mg (range 1.5–3.94 mg)	Austria	Rugg et al. [20]	Opioids were given for analgesia.
	6–10 years: 4.0 mg (range 3.0–7.50 mg)			
	11–14 years: 7.5 mg (range 3.75–7.5 mg)			
Morphine	0–5 years: 1.5 mg	Austria	Rugg et al. [20]	Opioids were given for analgesia.
	6–10 years: 4.0 mg (range 4.00–5.0 mg)			
	11–14 years: 5.0 mg (range 3.50–6.0 mg)			
	0–18 years: 0.05–0.1 mg/kg every 15 min	USA	Thomas et al. [23]	Was given for analgesia. Per protocol, “indicated for pain relief when a longer-acting agent is preferred and hemodynamic status is not at issue.”
Ketamine	0–16 years: 1.0 mg/kg (range 0.1–5.8 mg).	UK	Bredmose et al. [24]	Ketamine was given in sub-anesthetic dosages for painful procedure for analgesia and sedation, but it was difficult to separate the two effects.
Midazolam	0–16 years: 1 mg/kg (range 0.01–0.5 mg)	UK	Bredmose et al. [24]	The standard operating procedure for ketamine administration encourages co-administration of midazolam.
Esketamine	0–5 years: 15.0 mg (range: 10.0–25.0 mg)	Austria	Rugg et al. [20]	Esketamine was given for analgesia.
	6–10 years: 15.0 mg (range: 12.5–25.0 mg)			
	11–14 years: 20.0 mg (range: 15.0–25.0 mg)			

Schauer et al. [26] reported that only 618 (17.9%) of 3439 Afghan and Iraqi patients younger than 17 years of age who had military-related injuries received analgesia. This administration rate appears to be much lower than the reported 35–71% adults in similar settings [27,29]. Those authors emphasized that this low administration rate was nearly identical to that observed among adults prior to the implementation of the Tactical Combat Casualty Care guidelines [29], and that it highlights the need for similar recommendations for analgesia administration among pediatric patients [26,29].

### 3.3. ED Analgesia for Children with Major Trauma

There is only one publication on ED pain management in pediatric patients following multi-trauma [19]. Anantha et al. [19] explored the use of analgesia of severely injured children in the ED during the resuscitative phase and found that analgesia (81% parenteral) was provided to 32% (64/203) of severely injured children during the resuscitative phase. Forty-three of those patients (67%) were treated during the primary survey and twenty-one (33%) during the secondary survey. Of the analgesic group, narcotics were administered to almost 90% of the reported patients.

### 3.4. Pre-Hospital Medications

Curtis et al. [16] reported that the analgesia provided to the children before ED admission included morphine (20.5%), fentanyl (34.0%), ketamine (4.2%), and methoxyflurane (18.4%). Barker et al. [21] calculated that pain medications were administered to 54 (15%) patients, and that fentanyl and ketamine were used as analgesics rather than as sedatives. Rugg et al. [20] reported that 98% of the pediatric patients who received analgesics were treated with morphine or esketamine (the S-enantiomer of racemic ketamine). Most of the patients in their study were treated with an opioid or with esketamine as a monotherapy, and fewer with the combination of both drugs. The most frequently administered opioid was fentanyl, followed by piritramide and morphine. The preferred use of fentanyl over piritramide was the most evident in the youngest age group (i.e., 0–5 years of age). Schauer et al. [26] determined that morphine was the most frequently used drug in the military setting, accounting for 46.2% of all administrations of analgesia, followed by fentanyl (30.4%) and ketamine as an analgesic agent (17.4%). Most administrations of fentanyl were carried out in the 10- to 14-year-old group. Interestingly, ketamine was more frequently utilized in patients with an ISS > 15.

#### 3.4.1. Safety

Several pre-hospital aero-medical studies explored the safety of analgesia administration in pediatric patients following major trauma [20,22,23,24,26]. Rugg et al. [20] reported that opioids and esketamine were the drugs of choice in injured children transported in an Austrian HEMS. HEMS in Austria is typically physician-staffed, and the type of analgesia was not determined according to any protocol. Their study examined the safety of opioids and esketamine in combination and not each individually. Safety was assessed by respiratory rates, oxygen saturation (SpO_2_), and Mainz Emergency Evaluation Score (MEES) scores. The MEES score is a dynamic scoring system for pre-hospital EM services that is used to evaluate the efficacy and quality of the pre-hospital interventions as calculated by the Glasgow Coma Scale, heart and respiratory rates, cardiac rhythm, pain, blood pressure, and peripheral SpO_2_ [30]. Those authors reported that nearly 66% of the study patients did not require any additional measures whatsoever, and that more than 33% received only oxygen supplementation during transport. However, because intubated patients were excluded from the study, the requirement of immediate on-site intubation may have been missed. An additional analysis in that study revealed that nearly 80% of the children who received analgesia and required on-site intubation were classified as high-level injury severity, and therefore intubation was most probably indicated by injury severity rather than medication side effects.

#### 3.4.2. Fentanyl Safety

DeVellis et al. [22] reviewed the 5.5-year safety record of a protocol-guided fentanyl administration to pediatric patients with major trauma undergoing HEMS transport in the USA. Flight records were analyzed for narrative information indicative of fentanyl-related side effects, such as the need for naloxone administration and assisted ventilation. No adverse effects from fentanyl were noted; no patient became hypotensive, the mean pre-fentanyl oxygen saturation was not significantly different from the post-fentanyl oxygen saturation, and no patient had a clinically significant saturation decrement following fentanyl. Thomas et al. [23] studied the frequency, safety, and efficacy of pre-hospital fentanyl analgesia for a 6-month period in adult and pediatric helicopter trauma scene transports. There was no overall difference between pre- and post-analgesia systolic blood pressures; specifically, there were no cases of fentanyl-related hypotension among the children, and the post-analgesia SpO_2_ did not drop below 90% in any of the patients. Those authors concluded that fentanyl was provided frequently with good effect and minimal cardiorespiratory consequences in those settings.

#### 3.4.3. Morphine Safety

In their study conducted in a combat setting, Schauer et al. [26] evaluated pre-hospital analgesia among pediatric patients (<18 years of age) admitted to United States and Coalition fixed-facility hospitals in Iraq and Afghanistan. They reported a patient injured by an explosive who had received naloxone after the administration of morphine and ultimately survived until hospital discharge. There are no other publications related to the safety of morphine administration in children with major trauma.

#### 3.4.4. Ketamine Safety

Bredmose et al. [24] retrospectively reviewed 164 pediatric patients with blunt trauma injury who received ketamine during HEMS transport. Ketamine was administered solely by physicians with anesthesia or emergency medicine training and mostly used for analgesia and procedural sedation in non-trapped awake victims of blunt trauma. Those authors observed that ketamine in sub-anesthetic doses provided excellent analgesia and sedation for painful procedures, but that it was difficult to separate the two effects. They concluded that intravenous ketamine was the preferred medication for on-the-scene pediatric analgesia. No significant complications were associated with ketamine use, and there was usually a clinical explanation related to the injuries (chest injury) rather than to the administration of ketamine in the few cases of oxygen desaturation. Finally, those authors emphasized the importance of pre-hospital medical team competence in advanced airway management techniques in order to deal with uncommon complications of drug administration [24].

### 3.5. Medications Administered in the ED

Anantha et al. [19] reported that opioids (specifically, morphine and fentanyl) were the most common analgesics administered to major trauma pediatric patients (85%) in the ED, followed by non-steroidal anti-inflammatory drugs (10%), ketamine (8%), and acetaminophen (5%). There are no reports on regional nerve blocks, and their use during the resuscitative phase in multi-system trauma patients is unclear [31].

#### Safety

Ananthe et al. [19] reported that both analgesic and non-analgesic groups had similar rates of complications, including hypotension and respiratory depression, which are the known side-effects of many analgesics. Additionally, all of their pediatric and adolescent patients required imaging, and all were followed by an inpatient pediatric trauma service, with no increased incidence of missed injuries in the analgesia group. Those authors concluded that careful and individualized use of analgesics in severely injured children and adolescents does not increase their morbidity or mortality. There are no publications on the safety of the administration of opioids or ketamine in children presenting to the ED with major trauma.

### 3.6. Sedation of Children with Major Trauma in the Pre-Hospital and ED Settings

Hemodynamically stable major trauma pediatric patients who are neurologically intact are usually not treated with tracheal intubation. They often require sedation for painful or distressful procedures during their stay in the ED [13], such as fracture reduction, wound debridement, burn wound care, or advanced imaging requiring immobilization [28,32]. The administration of sedation in these patients nevertheless carries the potential risk of apnea, aspiration, or hypotension [33].

#### 3.6.1. Pre-Hospital Sedation

Bredmose et al. [24] explored pre-hospital use of ketamine for procedural sedation and analgesia in awake non-trapped patients with blunt trauma. Again, it was difficult to separate the effects of analgesia from those of sedation. No significant complications were associated with ketamine use, and there was usually a clinical explanation related to the injuries (chest injury) rather than to the administration of ketamine in the small number of cases of oxygen desaturation. Those authors concluded that pre-hospital ketamine administration provided by physicians with anesthesia or an emergency medicine background was not associated with significant complications [24].

#### 3.6.2. ED Sedation

Bar Am et al. [13] evaluated ED sedation-related adverse events in 455 non-intubated hemodynamically stable children with GCS 15 following severe trauma (ISS >15). Those authors reported that most of the patients were sedated with intravenous midazolam alone (27.4%), followed by combined intravenous midazolam and fentanyl (25.4%), and combined midazolam and ketamine (21.4%). All of the 201 reported procedures (computerized tomography, wound debridement, and fracture reduction) were completed without any severe adverse events. Of note, there were two (1%) cases of unanticipated endotracheal intubation due to sedation; those patients recovered uneventfully, and all 455 patients survived hospital stay. No cases of cardiopulmonary resuscitation during sedation or during hospital stay, and no cases of neurologic sequelae on hospital discharge were recorded among the patients who were treated with procedural sedation. Those authors concluded that non-intubated children with severe trauma who are hemodynamically stable and have a GCS of 15 are at low risk for severe adverse events due to sedation in a trauma unit where physicians are skilled in pediatric sedation, stressing that proficiency in pediatric airway management is a crucial skill for physicians involved in the sedation of these patients [13]. The benefit of sedation may outweigh the potential harm of an adverse event that can be effectively dealt with by a skilled trauma team physician when extremely painful and distressing procedures are required [13].

## 4. Conclusions

The available literature indicates that a relatively low percentage of pediatric patients who sustain major trauma receive analgesia in pre-hospital and ED settings. Studies that address the administration of sedation are sparse, and those that have been published highlight the importance of physician competency in managing pediatric airways. Prospective studies that focus upon the benefits and risks of providing analgesia and sedation to children who have sustained major physical trauma are warranted.

## Data Availability

Not applicable.

## Abbreviations

ED	Emergency department
HEMS	Helicopter Emergency Medical Services
ISS	Injury severity score
GCS	Glasgow coma scale
PSA	Procedural sedation and analgesia
ICU	Intensive care unit
MEES	Mainz Emergency Evaluation Score
BP	Blood pressure

## References

[1] Ward S.E., Gordon D. (1994). Application of the American Pain Society quality assurance standards. Pain.

[2] Helmerhorst G.T., Teunis T., Janssen S.J., Ring D. (2017). An epidemic of the use, misuse and overdose of opioids and deaths due to overdose, in the United States and Canada: Is Europe next?. Bone Jt. J..

[3] Williams K., Saeed N., Ihnow S., Mangeot C., Denning J. (2022). Spica Casting of Pediatric Femur Fractures: The Pain Management Experience at One Institution. Cureus.

[4] Atkinson P., Chesters A., Heinz P. (2009). Pain management and sedation for children in the emergency department. BMJ.

[5] Pardesi O., Fuzaylov G. (2017). Pain Management in Pediatric Burn Patients: Review of Recent Literature and Future Directions. J. Burn. Care Res..

[6] Ali S., Rajagopal M., Klassen T., Richer L., McCabe C., Willan A., Yaskina M., Heath A., Drendel A.L., Offringa M. (2020). Study protocol for two complementary trials of non-steroidal or opioid analgesia use for children aged 6 to 17 years with musculoskeletal injuries (the No OUCH study). BMJ Open.

[7] Greenwald M. (2010). Analgesia for the pediatric trauma patient: Primum non nocere?. Clin. Pediatr. Emerg. Med..

[8] Hildenbrand A.K., Kassam-Adams N., Barakat L.P., Kohser K.L., Ciesla J.A., Delahanty D.L., Fein J.A., Ragsdale L.B., Marsac M.L. (2020). Posttraumatic Stress in Children After Injury: The Role of Acute Pain and Opioid Medication Use. Pediatr. Emerg. Care.

[9] Hildenbrand A.K., Marsac M.L., Daly B.P., Chute D., Kassam-Adams N. (2016). Acute Pain and Posttraumatic Stress after Pediatric Injury. J. Pediatr. Psychol..

[10] Zohar Z., Eitan A., Halperin P., Stolero J., Hadid S., Shemer J., Zveibel F.R. (2001). Pain relief in major trauma patients: An Israeli perspective. J. Trauma Acute Care Surg..

[11] Buduhan G., McRitchie D.I. (2000). Missed injuries in patients with multiple trauma. J. Trauma Acute Care Surg..

[12] Beaulieu-Jones B.R., Bingham S., Rhynhart K.K., Croitoru D.P., Singleton M.N., Rutman M.S., Baertschiger R.M. (2022). Incorporating a Trauma-Informed Care Protocol into Pediatric Trauma Evaluation: The Pediatric PAUSE Does Not Delay Imaging or Disposition. Pediatr. Emerg. Care.

[13] Bar Am N., Samuel N., Ben-Lulu H., Bahouth H., Shavit I. (2019). Procedural sedation in non-intubated children with severe trauma—A single center study. Am. J. Surg..

[14] American College of Surgeons Committee on Trauma (2018). Advanced Trauma Life Support Student Course Manual.

[15] (2001). Injury Surveillance Guidelines.

[16] Curtis K., Kennedy B., Lam M.K., Mitchell R.J., Black D., Burns B., Loudfoot A., Tall G., Dinh M., Beech C. (2020). Prehospital care and transport costs of severely injured children in NSW Australia. Injury.

[17] Neighbor M.L., Honner S., Kohn M.A. (2004). Factors affecting emergency department opioid administration to severely injured patients. Acad. Emerg. Med..

[18] Silka P.A., Roth M.M., Geiderman J.M. (2002). Patterns of analgesic use in trauma patients in the ED. Am. J. Emerg. Med..

[19] Anantha R.V., Stewart T.C., Rajagopalan A., Walsh J., Merritt N.H. (2014). Analgesia in the management of paediatric and adolescent trauma during the resuscitative phase: The role of the pediatric trauma centre. Injury.

[20] Rugg C., Woyke S., Ausserer J., Voelckel W., Paal P., Ströhle M. (2021). Analgesia in pediatric trauma patients in physician-staffed Austrian helicopter rescue: A 12-year registry analysis. Scand. J. Trauma Resusc. Emerg. Med..

[21] Barker C.L., Weatherall A.D. (2014). Prehospital paediatric emergencies treated by an Australian helicopter emergency medical service. Eur. J. Emerg. Med..

[22] De Vellis P., Thomas S.H., Wedel S.K., Stein J.P., Vinci R.J. (1998). Prehospital fentanyl analgesia in air-transported pediatric trauma patients. Pediatr. Emerg. Care.

[23] Thomas S.H., Rago O., Harrison T., Biddinger P.D., Wedel S.K. (2005). Fentanyl trauma analgesia use in air medical scene transports. J. Emerg. Med..

[24] Bredmose P.P., Grier G., Davies G.E., Lockey D.J. (2009). Pre-hospital use of ketamine in paediatric trauma. Acta Anaesthesiol. Scand..

[25] Johnson T.J., Schultz B.R., Guyette F.X. (2014). Characterizing Analgesic Use during Air Medical Transport of Injured Children. Prehosp. Emerg. Care.

[26] Schauer S.G., Arana A.A., Naylor J.F., Hill G.J., April M.D. (2018). Prehospital Analgesia for Pediatric Trauma Patients in Iraq and Afghanistan. Prehosp. Emerg. Care.

[27] Schauer S.G., Mora A.G., Maddry J.K., Bebarta V.S. (2017). Multicenter, prospective study of prehospital administration of analgesia in the U.S. Combat Theater of Afghanistan. Prehosp. Emerg. Care.

[28] Godfrey J., Choi P.D., Shabtai L., Nossov S.B., Williams A., Lindberg A.W., Silva S., Caird M.S., Schur M.D., Arkader A. (2019). Management of Pediatric Type I Open Fractures in the Emergency Department or Operating Room: A Multicenter Perspective. J. Pediatr. Orthop..

[29] Schauer S.G., Robinson J.B., Mabry R.L., Howard J.T. (2015). Battlefield analgesia: TCCC Guidelines are not being followed. J. Spec. Oper. Med..

[30] Hennes H.J., Reinhardt T., Otto S., Dick W. (1993). Die The preclinical efficacy of emergency care. A prospective study. Anaesthesist.

[31] Popovic J., Morimoto M., Wambold D., Blanck T.J., Rosenberg A.D. (2006). Current practice of ultrasound-assisted regional anesthesia. Pain Pract..

[32] Holmes J.F., Mao A., Awasthi S., McGahan J.P., Wisner D.H., Kuppermann N. (2009). Validation of a prediction rule for the identification of children with intra-abdominal injuries after blunt torso trauma. Ann. Emerg. Med..

[33] Miner J.R., Martel M.L., Meyer M., Reardon R., Biros M.H. (2005). Procedural sedation of critically ill patients in the emergency department. Acad. Emerg. Med..

